# Early development of infant gut microbiota in relation to breastfeeding and human milk oligosaccharides

**DOI:** 10.3389/fnut.2023.1003032

**Published:** 2023-03-09

**Authors:** Maciej Chichlowski, Janna A. van Diepen, Andrei Prodan, Laurentya Olga, Ken K. Ong, Guus A. M. Kortman, David B. Dunger, Gabriele Gross

**Affiliations:** ^1^Medical and Scientific Affairs, Reckitt/Mead Johnson Nutrition Institute, Evansville, IN, United States; ^2^Medical and Scientific Affairs, Reckitt/Mead Johnson Nutrition Institute, Nijmegen, Netherlands; ^3^NIZO Food Research BV, Ede, Netherlands; ^4^Department of Paediatrics, University of Cambridge, Cambridge, United Kingdom; ^5^MRC Epidemiology Unit, Wellcome Trust-MRC Institute of Metabolic Science, University of Cambridge, Cambridge, United Kingdom; ^6^Wellcome Trust-MRC Institute of Metabolic Science, University of Cambridge, Cambridge, United Kingdom

**Keywords:** microbiome, human milk oligosaccharides (HMO), bifidobacteria, fucosyllactose, clinical

## Abstract

**Background:**

Infant gut microbiota composition is influenced by various factors early in life. Here, we investigate associations between infant gut microbiome development, infant age, breastfeeding duration, and human milk oligosaccharides (HMO) composition in breastmilk.

**Methods:**

A total of 94 mother-infant pairs were recruited as part of the Cambridge Baby Growth and Breastfeeding Study (CBGS-BF) (Cambridge, UK). Infant stool samples (*n* = 337) were collected at 2 week, 6 week, 3 month, and 6 month of age. The 16S rRNA V3-V4 rRNA region was sequenced using MiSeq Illumina to determine microbiota composition and diversity. Mother’s hindmilk samples were collected at birth, 2 week, 6 week, 3 month, and 6 month postpartum. Concentrations of five neutral [2′FL, 3′FL, lacto-N-fucopentaose 1 (LNFP1), LNnT, LNT] and two acidic (3′SL, and 6′SL) HMOs were measured in all milk samples using High-Performance Anion-Exchange Chromatography with Pulsed Amperometric Detection (HPAEC-PAD). We explored the associations between infant gut microbiome parameters and age, duration of exclusive breastfeeding (EBF), and levels of individual HMOs.

**Results:**

*Bifidobacterium* was the most abundant genus in infant stool at all-time points, irrespective of breastfeeding duration, with an overall mean relative abundance of 70%. The relative abundance of *B. bifidum* in stool from infants who were breastfed for longer than 6 months was significantly higher compared to the infant breastfed up to 3 months (*p* = 0.0285). Alpha-diversity (both Shannon and ASV-level Richness) of infant gut microbiota showed a biphasic change with infant age, decreasing from 2 weeks until 3 months and then increasing until 6 months of age. *Bifidobacterium* relative abundance was associated with higher concentrations of 2′FL and LNFP1 in breastmilk across all time-points (*p* = 0.049 and 0.017, respectively), with trends toward a higher abundance of *B. longum* species. No significant association with *Bifidobacterium* was found for breastmilk LNnT, 3′SL, and 6′SL levels.

**Conclusion:**

Our study is in line with previous data demonstrating that EBF duration in the first months of life impacts infant gut microbiota composition. The observed links between specific HMOs in breastmilk and bacteria in infant stool provide evidence of how mother’s milk affects infant microbiome development.

## Key message and impact

Our study demonstrates that exclusive breastfeeding during the first months of life significantly impacts infant gut microbiota composition. In a cohort of healthy mother-infant pairs in the UK we observed a trend of decreased microbial diversity in infant fecal microbiota during the first 3 months of age. It was followed by an increased diversity at 3 months of age, which coincides with prominent changes in human milk oligosaccharides (HMO) concentrations in corresponding breastmilk samples from respective mothers during the first 3 months. In our study, select bifidobacteria levels in infant stool were higher in infants who were exclusively breastfed longer (over 6 months) compared to infants breastfed for a shorter period of time (less than 3 months). These associations between levels of specific HMOs in breastmilk and bacteria in infant stool provide important insights into how mother’s milk affects infant microbiome development.

## Introduction

Gut microbiota plays a critical role in influencing infant growth, body composition, and later life health *via* modulating gastrointestinal, nervous, and immune systems, as well as energy metabolism and fat deposition (1). Infant gut microbiota develops and evolves in the first 1,000 days of life and would attain adult-like composition by the age of 3 years (2). In addition to *Bifidobacterium* spp., anaerobic bacteria such as *Bacteroides* spp. and *Clostridium* spp. have also been identified as colonizers of infant gut during the first 6 months of life and are known to have various effects on infant development and maturation (3).

Infant feeding has been established as one of the major factors shaping he development of gut microbiota (4). Gut microbiome and fecal metabolites of breastfed infants change during lactation and are influenced by breast milk components (5); breastmilk promotes the selective proliferation of a characteristic microbiota. Bacterial families in exclusively breastfed infants have a higher occupancy of *Bifidobacteriaceae* and a lower presence of *Enterococcaceae* and *Enterobacteriaceae* than those in formula-fed infants (6). However, bacterial genera such as *Bifidobacterium* spp. fluctuate dramatically in exclusively breastfed infants (7, 8), which may be partly explained by mother’s variation in breastmilk (BM) composition.

Human milk oligosaccharides (HMOs) support bifidobacteria-predominant gut microbiota in breastfed infants (9). HMOs represent the third most abundant solid human milk component after lipid and lactose, even higher than protein (10). With a concentration of 5–10 g/L, HMOs make BM distinct from the milk of other farm animals, including cows, whose oligosaccharide concentrations are 100–1,000-fold lower (10). HMOs composition in BM depends on several factors, including the maternal secretor (*FUT2*) genotype. Fucosylated lactoses such as 2′-fucosyllactose (2′FL), sialylated lactoses such as 3′-sialyllactose (3′SL) and 6′SL, and oligosaccharides with lacto-N-biose structure such as lacto-N-fucopentaose (LNFP) 1 are some of the most abundant HMOs in BM.

HMOs act as selective prebiotics for the gut microbiota (11), and associations between HMOs and infant gut microbiome development have been demonstrated. *Bifidobacterium longum* subsp. *infantis* (*B. infantis*) is the most well-known species that is particularly well-suited to colonize the infant gut (12), attributed to its unique ability to consume a large variety of HMOs. As evidenced in a clinical study (13), the administration of *B. infantis* may lead to successful colonization in the gut, whereby its highly selective and acidic fermentation of HMOs increases the production of lactate and acetate and subsequently reduces intestinal pH as well as counteracts gut dysbiosis [reviewed previously (14)]. This potentially includes an important role in the development and maturation of the immune system. Although HMOs are also consumed by other bacteria (e.g., *Bacteroidaceae*), in general, *Bifidobacteriaceae* is the only bacterial family that can substantially convert HMOs to acidic end products that affect stool pH (15). Previously, Bai et al. reported changes in HMOs in the milk of Chinese mothers with different secretor statuses during 6 months of lactation. Those researchers observed the correlations between fucosylated HMOs (e.g., 2′FL) and *Bifidobacterium* spp. in infant stool during early lactation (16). The impact of milk composition on infant stool microbiome was also suggested by Pace and colleagues, who focused on HMO analyses in different geographical locations (17). Finally, Borewicz and colleagues investigated the associations between concentrations of selected HMOs and infant stool microbiota in 24 mother-infant pairs at 2, 6, and 12 weeks of age. Microbiota composition was associated with mode of delivery and breastmilk LNFP1, 3′SL, and Lacto-N-hexaose (LNH) at different time points (18). Despite accumulating knowledge on the effect of HMOs on the infant gut microbiome [summarized elegantly in a recent review by Masi and Stewart (19)], clinical and cohort studies confirming associations between HMOs levels and infant gut microbiota composition in infant-mother cohorts are still scarce.

In the present analysis, we focused on investigating the associations between early gut microbiota development and composition with infant age, exclusive breastfeeding (EBF) duration as well as individual HMOs by analyzing breastmilk and stool samples during the first 6 months of lactation from 94 mother-infant pairs of the Cambridge Baby Growth and Breastfeeding Study (CBGS-BF) (Cambridge, UK).

## Materials and methods

### Study design and participants

**FIGURE 1:**
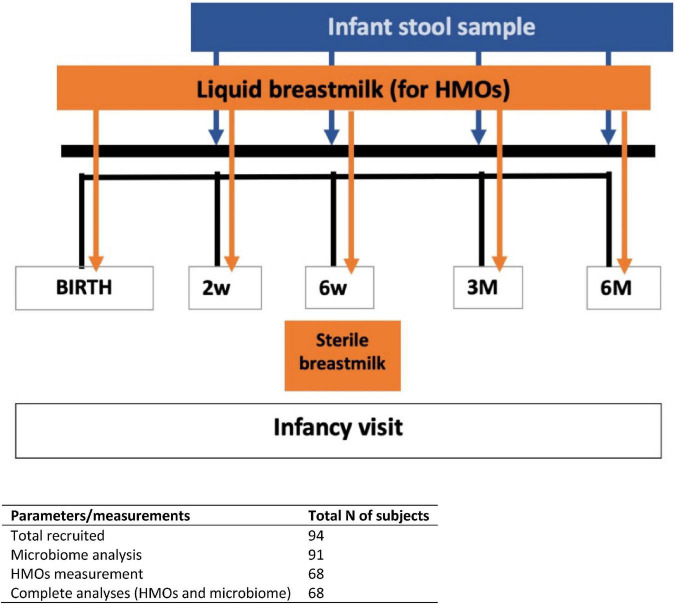
Timeline for the entire study duration. Fecal and breast milk samples were collected based on specified days (infant’s age) as stated for each visit.

This study is part of the CBGS-BF, a UK-based prospective observational infant cohort (20). The CBGS-BF is an extension of the original Cambridge Baby Growth Study (21), aiming to investigate determinants of infant growth and body composition. All infants recruited to this cohort were singletons and vaginally born at term from healthy mothers with normal pre-pregnancy BMI and without any significant comorbidities (*n* = 94) (20). All infants received EBF for at least 6 weeks. Infant stool samples were collected at 2 week, 6 week, 3 month, and 6 month (Figure 1). Mother milk samples were collected at birth, 2 weeks, 6 weeks, 3 month, and 6 month postpartum. The study was approved by the Cambridge Local Research Ethics Committee, and all mothers gave written informed consent.

### Feeding modules

For statistical analysis, infants were categorized into three feeding groups based on the duration of EBF:

1. Less than 3 months (< 3 month EBF), these infants received EBF for at least 6 weeks.

2. 3–6 months (3–6 month EBF).

3. More than 6 months (> 6 month EBF).

### Human milk oligosaccharides analysis

Levels of selected HMOs in maternal breast milk samples were quantitatively determined using high-performance anion-exchange chromatography as reported by Durham et al. (22). The analyzed HMOs included: 2′FL, 3′FL, LNFP1, LNnT, LNT, 3′SL, and 6′SL. For generalized linear mixed models (gLMM) “low” or “high” HMO levels were defined as lower or higher than median levels at each time point).

### Infant gut microbiome analysis

Detailed methodology describing DNA extraction from stool samples is provided in the Supplementary material. Briefly, 250 mg of fecal sample in S.T.A. R. Buffer (Roche, Indianapolis, IN, USA) were lysed with glass beads. After centrifugation, the supernatant was kept on ice. Purification of DNA was performed on the automated Maxwell instrument (Promega, Madison, WI, USA) by applying the Maxwell 16 Tissue LEV Total RNA Purification Kit (Promega) following the manufacturer’s protocol.

### PCR amplification of 16S rRNA gene in infant stool DNA, library preparation and sequencing

After DNA extraction, PCR amplification and DNA sequencing of the V3-V4 region of the bacterial 16S rRNA gene was performed in an Illumina MiSeq instrument (Illumina, San Diego, CA, USA). Detailed methodology describing PCR amplification and sequencing is provided in the Supplementary material.

### Data analysis

A detailed description of analytical methods is provided in the Supplementary material. Shannon index and ASV (amplicon sequence variant)-level richness were calculated using the vegan v2.5-7 R package (23). Faith’s phylogenetic diversity was calculated using the picante v1.8.2 R package. Weighted Unifrac distances were calculated using the phyloseq UniFrac function. PERMANOVA analyses were performed on the fecal microbiome weighted Unifrac distance matrixes using the adonis function of the vegan R package with 10,000 permutations to estimate the proportion of beta-diversity explained by time point, feeding module, or their interaction, For exclusively breastfed infants only, the effect of individual HMOs—2′FL, 3′FL, LNT, LNnT, LNFP1, 3′SL, and 6′SL—on microbial composition were assessed using PERMANOVA models of weighted UNIFRAC distances at each time point.

Beta-diversity was visualized using Principal Coordinate Analysis (PCoA) performed with the ape v5.5 R package (24) on weighted Unifrac distances.

Generalized linear mixed models (gLMM) were fitted using glmmTMB v1.7.22. Multiple comparison adjustment was performed using the False Discovery Rate (FDR) controlling procedure of Benjamini-Hochberg to limit the FDR to 5% (25). The DHARMa, performance and parameters R packages were used for model diagnostics. All statistical modeling and visualizations were performed in R v4.1.1 using the tidyverse v1.3.1 package. The metacoder v0.3.5 R package was used to create heat tree visualizations. The effect of the individual infant was modeled as a random effect, and an offset term was added to account for differences in library size (i.e., sequencing depth) between the samples.

## Results

Data was collected from 91 infants and 68 mothers. Infant sex (35 females, 56 males), parity (1 for 35 infants, 2 for 47 infants, 3 for 7 infants, and 4 for 2 infants), and FUT2 secretory status (56 producers, 12 non- or low producers) were included in the analysis as covariates.

Infants included in the analysis were classified based on EBF duration: < 3 months EBF (*n* = 13), 3–6 months EBF (*n* = 51), and > 6 months EBF (*n* = 27; see *Feeding Modules* above).

### Infant stool microbiome composition and diversity over time and per feeding module

**FIGURE 2:**
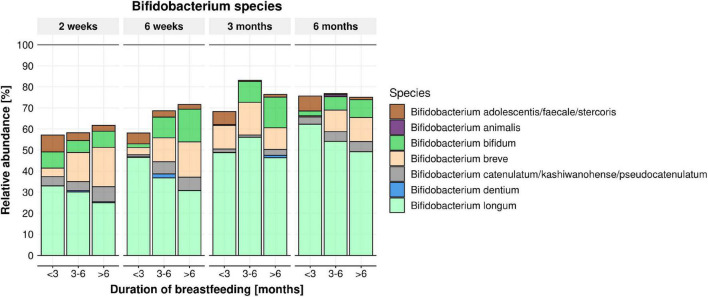
Mean relative abundance of *Bifidobacterium* species per feeding module (breastfeeding duration < 3, 3–6, > 6 months) across time (infant age 2 weeks, 6 weeks, 3 months, 6 months). *B. bifidum* abundance was significantly impacted by feeding modules (higher in infants breastfed > 6 month. compared to infants breastfed < 3 month; within the 3–6 months’ time frame) (*p* = 0.0285).

**FIGURE 3:**
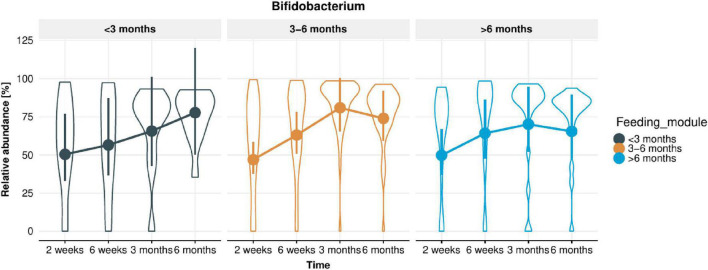
Change in *Bifidobacterium* relative abundance in infant stool microbiota from 2 weeks to 6 months of infant age in each of the feeding modules (breastfeeding duration < 3, 3–6, > 6 months). Overall, *Bifidobacterium* relative abundance increased significantly over time (*p* < 0.001).

From 16S rRNA gene sequencing, a total of 1,037 ASVs were detected in the infant stool samples. The fifteen most abundant taxa per feeding module and infant age are shown at genus level (Supplementary Figure 1A) and species level (Supplementary Figure 1B). Irrespective of breastfeeding duration, the genus *Bifidobacterium* was the most abundant taxon at 2 weeks of infant age (∼50% of infant gut composition) and increased further to ∼75% of total microbiome composition at 6 months of age (Figure 2; *p* < 0.001). *Bifidobacterium bifidum* abundance was significantly associated with EBF duration (higher in infants EBF > 6 month than EBF < 3 month; *p* = 0.0285). In addition, the abundance of *Enterococcus durans/faecalis/faecium* was lower in both longer EBF groups compared to infants EBF < 3 month (Supplementary Figure 1B). Changes in *Bifidobacterium* relative abundance from 2 weeks to 6 months are shown in Figure 3, with levels increasing from 2 weeks to 3 month for all three EBF groups. Thereafter, the levels of *Bifidobacterium* decreased between 3 and 6 month among infants who remained EBF (EBF 3–6 and > 6 month).

**FIGURE 4:**
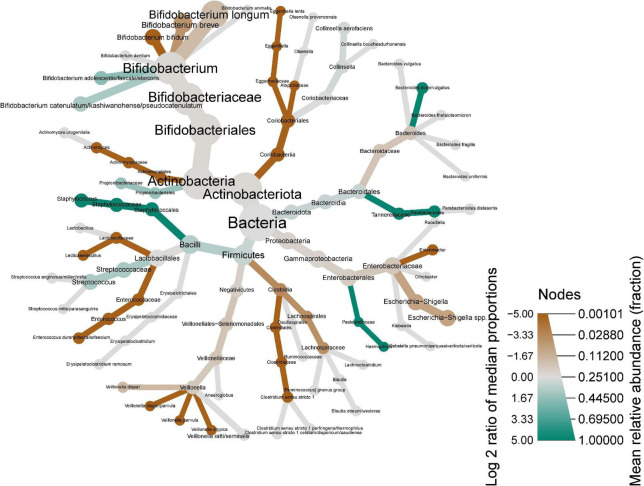
Heat tree showing shifts in infant stool microbiome composition between 2 weeks and 6 months of infant age. Nodes represent each taxonomic rank from kingdom (bacteria, center) species (tips of each branch). Node color indicates the log2 of the median proportions of each taxa at the two time points (i.e., green shows an increase, red shows a decrease), while the node size represents the respective taxon’s mean relative abundance.

The heat tree shown in Figure 4 provides a visual representation of the global changes in the infant fecal microbiome from age 2 weeks to 6 months. *B. adolescentis/faecale/stercosis* and *B. catenulatum/kashiwanohense/pseudocatenulatum* were higher at 6 months while other bifidobacteria (e.g., *B. bifidum*, *B. longum*, and *B. breve*) were lower.

**FIGURE 5:**
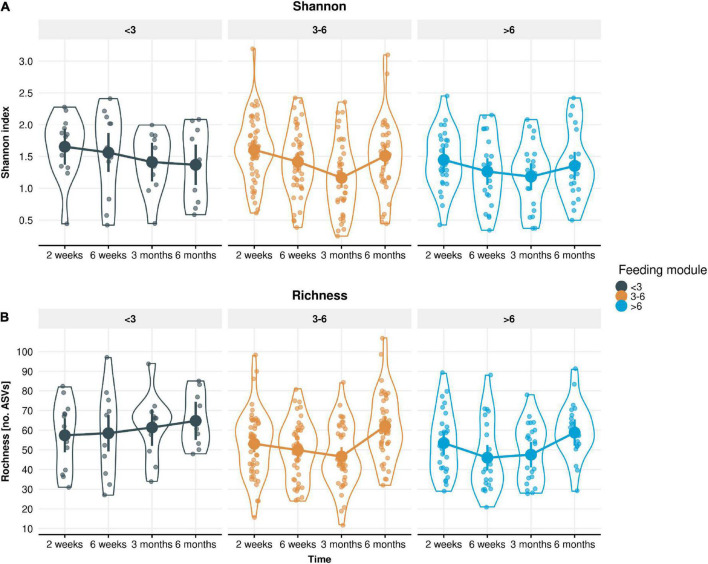
Measured alpha-diversity values displayed by **(A)** Shannon index and **(B)** Richness. LMM group estimates with 95% confidence intervals across the four time points (infant age 2 weeks, 6 weeks, 3 months, 6 months) stratified by the three feeding modules (breastfeeding duration < 3, 3–6, > 6 months). Richness was significantly lower in longer breastfed infant groups (*p* = 0.01 in the 6 weeks to 3 months period and *p* = 0.058 in the 3–6 months period).

The impact of EBF duration on stool microbiome diversity was also evaluated. A longitudinal trend of decreased alpha-diversity (both Shannon and ASV-level Richness) was observed from 2 weeks until 3 months of infant age (except for Richness in infants EBF < 3 month), followed by an increase at 6 months of age (Figure 5A). Regardless of EBF duration, Shannon index decreased by 0.13 units from 2 to 6 weeks (*p* = 0.0068), and a further 0.14 units from 6 weeks to 3 months (*p* = 0.0022), but then increased by 0.15 units from 3 to 6 months (*p* = 0.00026). Richness decreased by 2.97 ASVs from 2 to 6 weeks (*p* = 0.0022), was unchanged from 6 weeks to 3 months (*p* = 0.50), then increased by 8.48 ASVs from 3 to 6 months (*p* = 2.7 × 10–8). Moreover, Richness was significantly lower in longer EBF groups. This difference was significant around 12 ASVs in the 6 weeks to 3 months period (*p* = 0.01) and around 9 ASVs in the 3–6 months period (*p* = 0.058; Figure 5B). At 6 weeks, the estimated number of ASVs for infants in the < 3 month. breastfed group was 61 ASVs, while the estimate for the 3–6 months breastfed groups was 49 ASVs (lower for the > 6 month group). At 6 months, the estimated number of ASVs for infants in the < 3 month. breastfed group was around 70 ASVs, while the estimate for the longer breastfed groups was around 61 ASVs.

**FIGURE 6:**
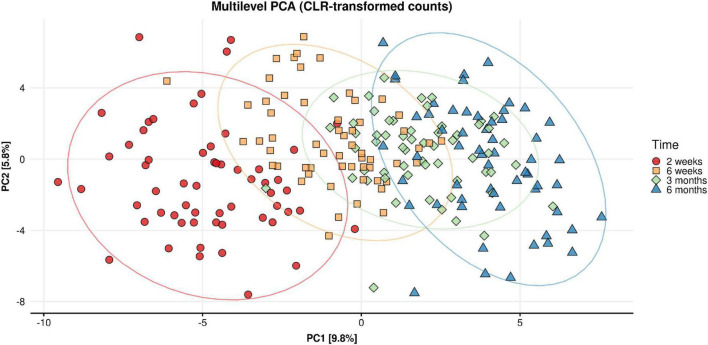
Multilevel principal component analysis plot of CLR-transformed microbial counts for infant stool microbiota composition across time (infant age 2 week, 6 weeks, 3 months, 6 months). Only intra-individual variance is decomposed and shown. Inter-individual variance (i.e., between infant differences) is removed.

Beta diversity analysis of the fecal microbiome showed that most of the variance in the weighted Unifrac (quantitative) distances was influenced by inter-individual differences (infants) (R2 = 0.556, *p* < 0.001) and time point (R2 = 0.06, *p* < 0.001). Figure 6 shows the Principal Component Analysis plot illustrating the intra-individual variance (after removing inter-individual variance) and demonstrates the shift in microbiota composition over time.

### Associations between HMOs levels in breastmilk and abundance of *Bifidobacterium* in infant gut

**FIGURE 7:**
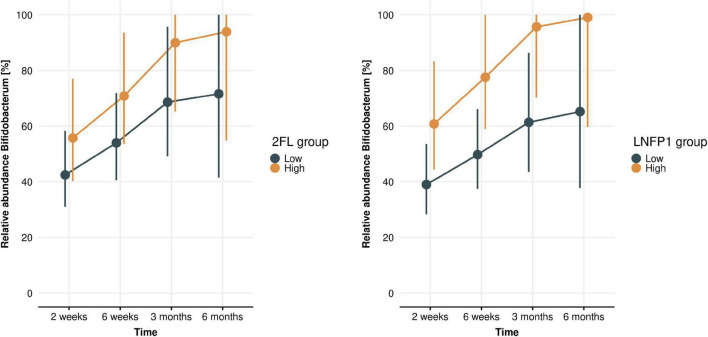
*Bifidobacterium* levels across time (infant age 2 weeks, 6 weeks, 3 months, 6 months) in high and low 2′FL **(left)** and LNFP1 **(right)** groups. Plots show gLMM estimates (95% confidence intervals) of *Bifidobacterium* relative abundance in infants with either low or high HMO levels (where “low” or “high” is defined as lower or higher than median levels at each time point). 2FL (*p* = 0.049) and LNFP1 (*p* = 0.017) were significantly associated with a higher *Bifidobacterium* relative abundance.

Associations between levels of individual HMOs and *Bifidobacterium* spp. relative abundance in infant gut microbiota were examined using gLMMs. Only samples collected while infants were exclusively breastfed were included in these models (*n* = 91 at 2 weeks, *n* = 79 at 6 weeks, *n* = 63 at 3 months, and *n* = 21 at 6 months). Over time (across all four time points), higher levels of 2′FL and LNFP1 in breastmilk (*p* = 0.049 and 0.017, respectively) were associated with higher *Bifidobacterium* relative abundance in infant stool (Figure 7), while a higher LNT level was associated with a lower relative abundance of *Bifidobacterium* (*p* = 0.029, Supplementary Figure 4A). LNnT, 3SL, and 6SL levels were not associated with *Bifidobacterium* relative abundance (Supplementary Figures 4B–D). Regarding *Bifidobacterium* species, we observed a trend toward higher *B. longum* levels with higher LNFP1 levels (*p* = 0.078). Associations between levels of individual HMOs and relative abundance of other bacterial groups described to metabolize HMOs were either not significant (*Bacteroides;* Supplementary Figure 5) or only present in ≤ 2 infant samples and therefore no statistical modeling was possible (*Akkermansia*, *Roseburia*, or *Eubacterium*).

## Discussion

Breast milk is ideally the nutritional source for infants since it contains various bioactive components, such as HMOs and antibacterial properties. However, relationships between human milk components, infant gut microbiome, and fecal metabolites throughout lactation remain largely unclear.

This analysis is part of the CBGS-BF recently described by Olga et al. (20). Durham and colleagues reported varying patterns of individual HMO concentrations longitudinally in the same cohort and concluded that the most prominent temporal changes occurred during the first 3 months (22). The present study aimed to analyze infant gut microbiota composition during the first 6 months of life and associations with EBF duration overall, as well as levels of HMOs in breastmilk that could potentially drive changes in microbial communities.

Our study has demonstrated that the duration of EBF significantly affected the infant gut microbiota during the first 6 months of life. We chose to visualize changes in microbiota data for each EBF feeding group separately, even at 2 and 6 weeks (before discontinuation of breastfeeding). This reflects the longitudinal data analysis that was performed. Another reason to separate the EBF feeding groups at 2 and 6 weeks is because infant characteristics (i.e., slower weight gain associated with reduced milk intake were seen in the group who stopped EBF earliest) in the EBF feeding groups already differ before discontinuation of EBF [previously published studies with the same cohort (20, 26)]. There were, however, no significant differences in alpha diversity or Bifidobacterium species between feeding groups at 2 weeks (data not shown).”

It has been reported that the gut microbiome of formula-fed infants is more diverse but less stable compared to breast-fed infants (27). In that study, alpha diversity was lower in breast-fed infants compared to formula-fed infants during the first 3 months after birth but increased significantly at 6 months of age. It has been suggested that higher bacterial diversity in formula-fed infants leads to a shift toward an adult-like microbiome at earlier ages (28). Lower diversity in infants receiving human milk is likely due to the clear dominance of *Bifidobacterium*, which preferentially utilize HMOs as substrates for growth. Komatsu and colleagues indicated that the gut microbiome of breastfed infants fluctuated after month 3 postpartum, even though milk components did not change (5). This likely indicates more efficient metabolism of breast milk components by bifidobacteria. Accordingly, it has been reported that after 3 months of age, the functional maturation of the infant GI tract progresses rapidly, and fecal properties change dramatically (29). In our study, *Bifidobacterium* was the most abundant genus in infant stool at all-time points and irrespective of EBF duration, with an overall mean relative abundance of 70%. Our data shows that levels of *Bifidobacterium* increased until 3 months of age for all infants but decreased during the period from 3 to 6 months of age in infants who were exclusively breastfed beyond 3 months. On the species level, infants EBF for > 6 months showed a higher relative abundance of *B. bifidum* compared to infants EBF for < 3 months. Analyzing the same cohort, Durham and colleagues reported varying longitudinal patterns of individual HMO concentrations and concluded that the most prominent temporal changes occur during the first 3 months (22). In line with the fluctuation of the dominant bifidobacteria, we here describe a biphasic longitudinal trend of initially decreasing alpha-diversity (both Shannon and ASV-level Richness) from 2 weeks until 3 months of infant age followed by an increase until 6 months of age. In addition, richness was lower in infants who were longer EBF.

In our study, levels of selected HMOs in breastmilk were significantly correlated with higher relative abundance of bifidobacteria in infant stool. In particular, levels of 2′FL and LNFP1 were positively correlated with higher bifidobacteria levels, from 2 weeks to 6 months, indicating that bioactive milk components, such as HMOs, contribute to shaping infant gut microbiota composition in exclusively breastfed infants. Another study recently reported positive correlations between HMOs (3′FL and 3′SL) and *B. breve* in infant stool (5). Here, we observed a trend toward higher abundance of *B. longum* in infant stool associated with higher levels of LNFP1 in breastmilk. We could not discriminate between subspecies *B. longum* subsp. *longum* and *B. longum* subsp. *infantis*, but the trend between LNFP1 levels and *B. longum* can likely be attributed most to *B. longum* subsp. *infantis. B. longum* subsp. *infantis* is equipped with fucosidases that are required to assimilate fucosylated HMOs, while *B. longum* subsp. *longum* generally is not (30). However, no associations were observed between *Bifidobacterium* (spp.) and LNnT, 3′SL, or 6′SL levels. The sialidases, required to utilize 3′SL and 6′SL (31), are also commonly detected in *B. longum* subsp. infantis, but not in *B. longum* subsp. *longum* (30, 32). Nonetheless, specific *Bifidobacterium* species that thrive on specific HMOs would particularly benefit from higher levels of these HMOs in breastmilk, resulting in higher relative abundance. HMO metabolism by *Bifidobacterium* species has been studied mechanistically, in particular using *in vitro* bacterial cultures allowing in depth analyses of specific bacterial strains or consortia and bacterial genes responsible for HMO utilization patterns (33, 34). However, evidence from clinical and cohort studies are limited so far to confirm associations between levels of specific HMOs in breastmilk and abundance of bacterial species in infant gut microbiota. To our knowledge, our present study is one of only few to explore natural variation in HMO levels and its impact on infant gut microbiota composition (35).

Our results provide direct evidence that the infant gut microbiome is dynamically associated with levels of bioactive milk components during a certain lactation period. Since gut microbiota in this sensitive time window in early life plays an important role in the infant’s physiology and development of the immune system (36), these insights are important when aiming to support the best start in life and ultimately optimal health outcomes at later ages.

## Study limitations

In this study, the microbiome profiling methodology (16S rRNA gene sequencing) did not allow to distinguish subspecies of *B. longum*, therefore we cannot determine the impact of select bioactives on *B. infantis* spp. Next, only a select panel of seven HMOs was analyzed due to lack of reliable standards at time of analysis. On top of the current analysis, it would be of interest to analyze the effects of actual HMO intake instead of breastmilk levels on infant gut microbiota composition. Breastmilk intake volume was only assessed at limited timepoints in the current study. Therefore, analysis on HMO intake would have been underpowered and was not performed.

## Conclusion

Our study demonstrates that EBF duration during the first months of life impacts infant gut microbiota composition. *Bifidobacterium* was the most abundant genus in infant stool at all-time points and irrespective of breastfeeding duration, with an overall mean relative abundance of 70%. *B. bifidum* levels were higher in infants who were breastfed longer (over 6 months) compared to infants breastfed for a shorter period of time (less than 3 months). In addition, richness was significantly lower in the longer breastfed infant groups across time points. Links between specific HMOs in breastmilk and bacteria in infant stool as demonstrated in this study provide evidence for how components present in mother’s milk affect infant microbiome development.

## Data availability statement

The raw sequencing data presented in this study are deposited in the European Nucleotide Archive accession number PRJEB50418.

## Ethics statement

The study was conducted according to the guidelines of the Declaration of Helsinki and approved by the National Research Ethics Service Cambridgeshire 2 Research Ethics Committee (IRAS No 67546, REC No 11/EE/0068).

## Author contributions

GG, JD, MC, LO, KO, and DD conceived and designed the study. AP and GK conducted the microbiome analysis. MC drafted the manuscript. All authors interpreted the data, contributed to manuscripts drafts, reviewed, and revised the manuscript for critical intellectual content, and approved the final version to be published.
