# Supplementary material for: Early development of infant gut microbiota in relation to breastfeeding and human milk oligosaccharides
Source: Front Nutr. 2023 Mar 9;10:1003032. doi: 10.3389/fnut.2023.1003032 (PMC10034312; doi:10.3389/fnut.2023.1003032)
Supplement: Supplementary file 1 [file Image_1.pdf]

Supplementary Figures

Suppl Fig 1.

a)

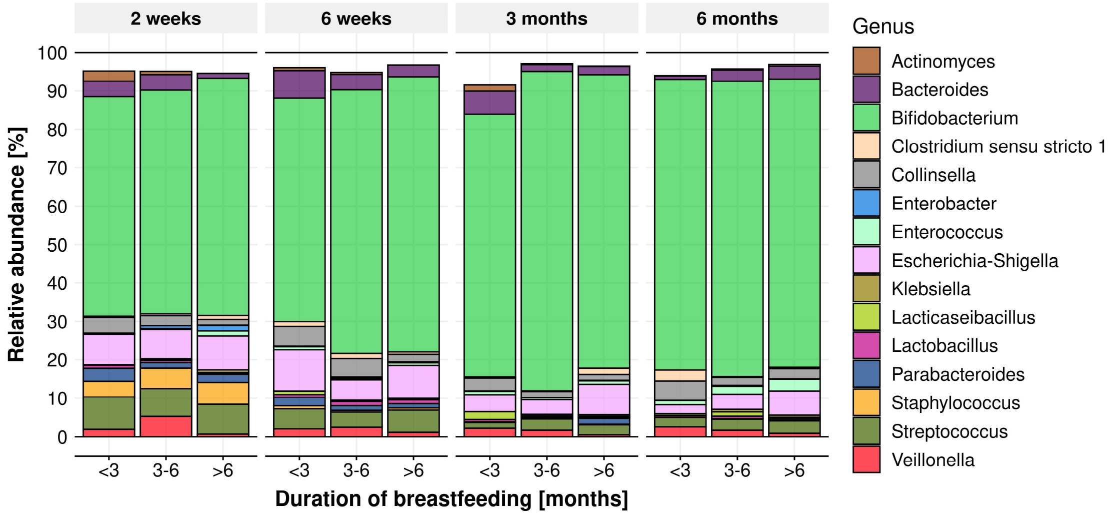

Suppl Fig 1

b)

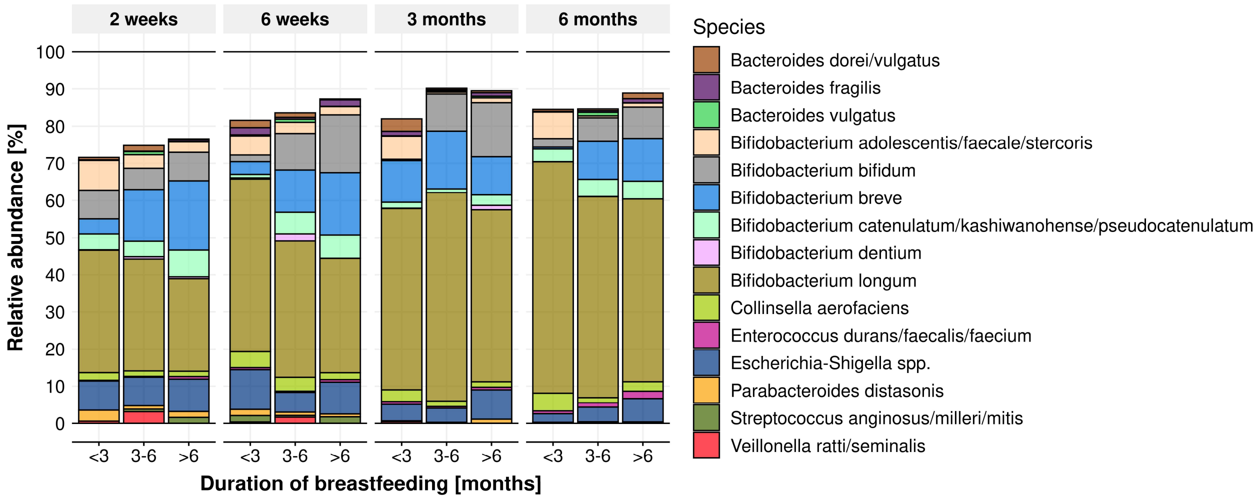
2 weeks
6 weeks
3 months
6 months
Relative abundance [%]
100
90
80
70
60
50
40
30
20
10
0
<3
3-6
>6
Duration of breastfeeding [months]
Species
Bacteroides dorei/vulgatus
Bacteroides fragilis
Bacteroides vulgatus
Bifidobacterium adolescentis/faecale/stercoris
Bifidobacterium bifidum
Bifidobacterium breve
Bifidobacterium catenulatum/kashiwanohense/pseudocatenulatum
Bifidobacterium dentium
Bifidobacterium longum
Collinsella aerofaciens
Enterococcus durans/faecalis/faecium
Escherichia-Shigella spp.
Parabacteroides distasonis
Streptococcus anginosus/milleri/mitis
Veillonella ratti/seminalis

Suppl Fig 2.

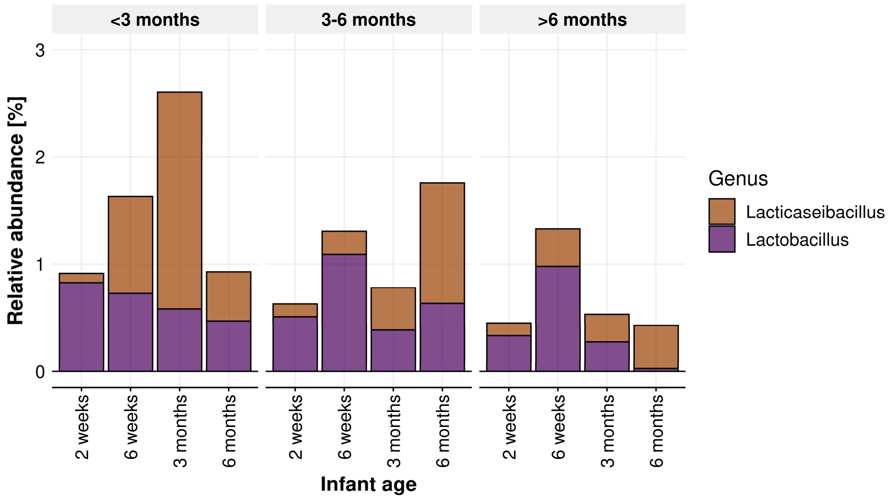

<3 months
3-6 months
>6 months
Relative abundance [%]
3
2
1
0
2 weeks
6 weeks
3 months
6 months
Infant age
Genus
Lacticaseibacillus
Lactobacillus

Suppl Fig 3.

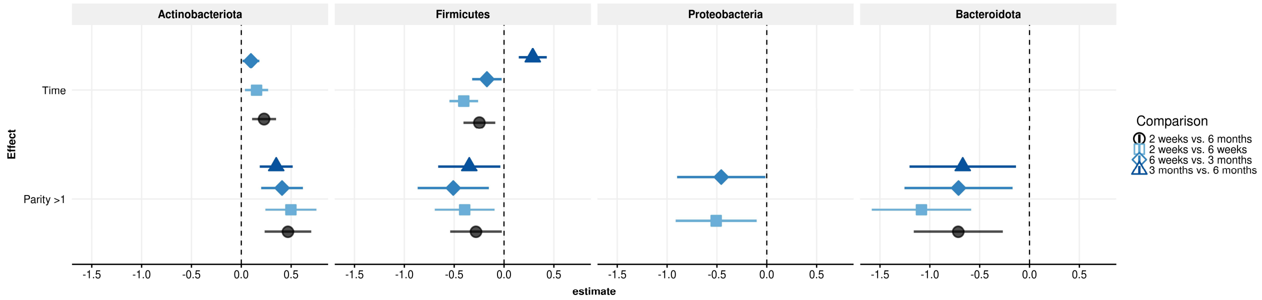
Actinobacteriota
Firmicutes
Proteobacteria
Bacteroidota
Time
Parity >1
Effect
estimate
-1.5
-1.0
-0.5
0.0
0.5
Comparison
2 weeks vs. 6 months
2 weeks vs. 6 weeks
6 weeks vs. 3 months
3 months vs. 6 months

Suppl Fig 4

a)

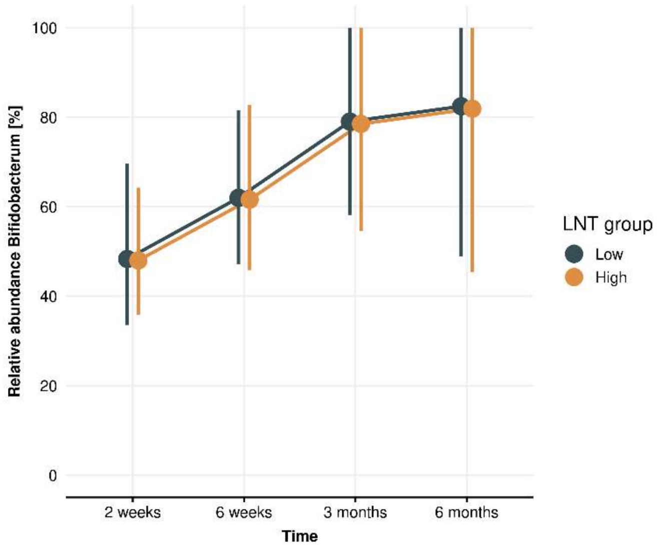

b)

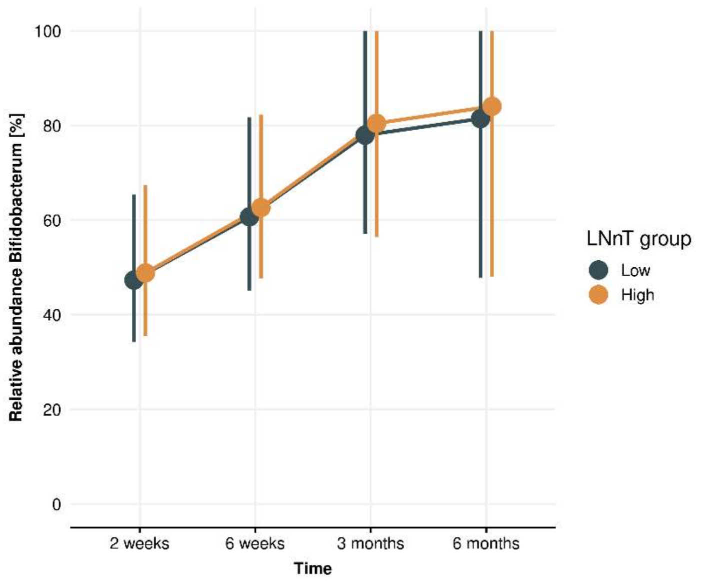

c)

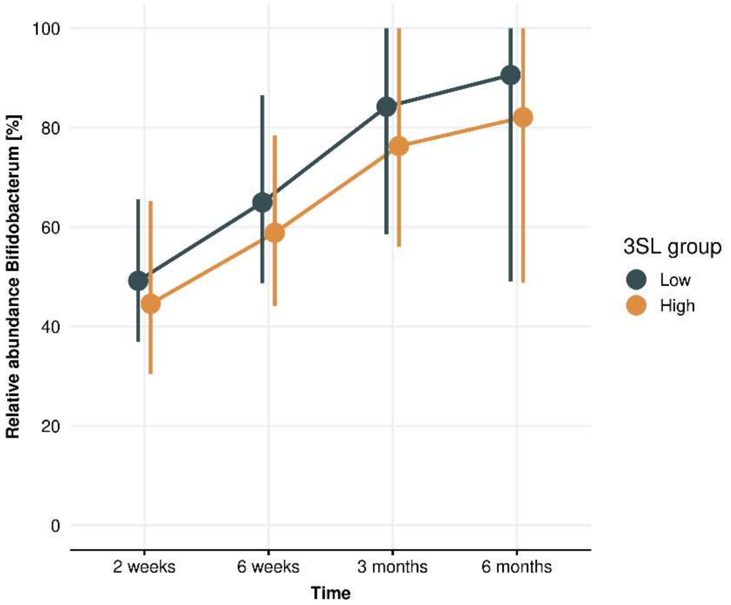
100
80
60
40
20
0
Relative abundance Bifidobacterium [%]
2 weeks
6 weeks
3 months
6 months
Time
3SL group
Low
High

d)

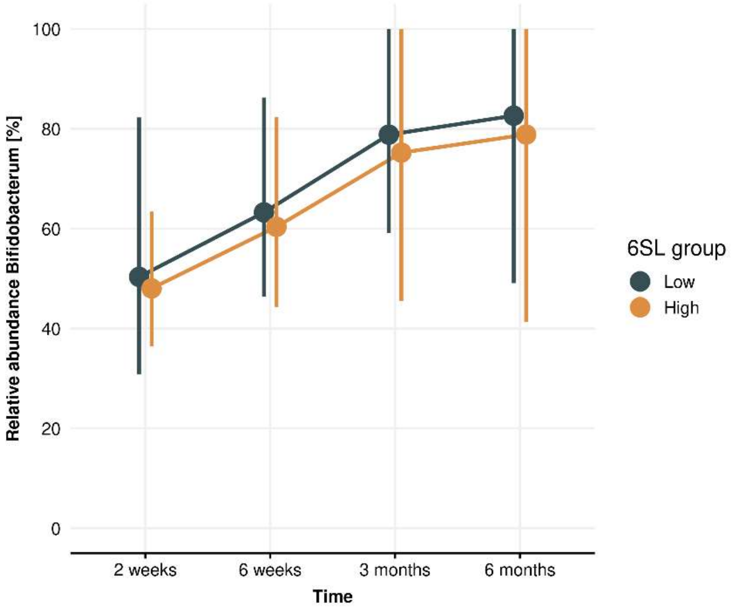
100
80
60
40
20
0
Relative abundance Bifidobacterium [%]
2 weeks
6 weeks
3 months
6 months
Time
6SL group
Low
High

Suppl Fig 5

a)

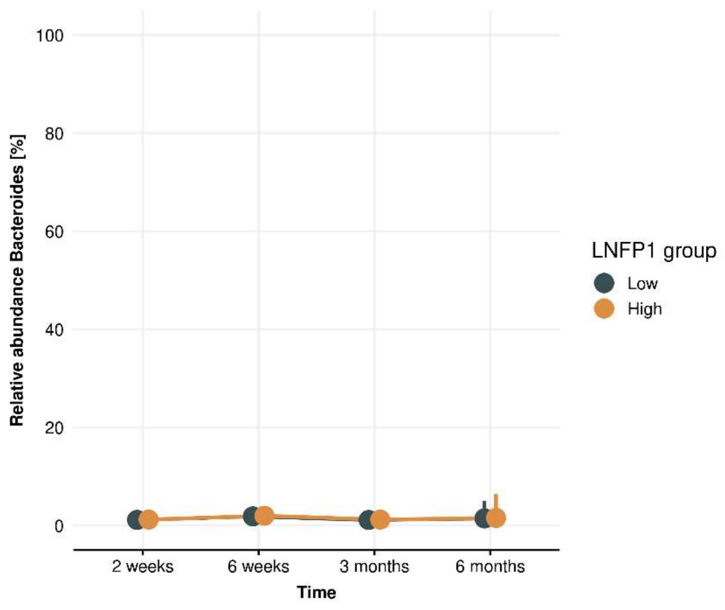

b)

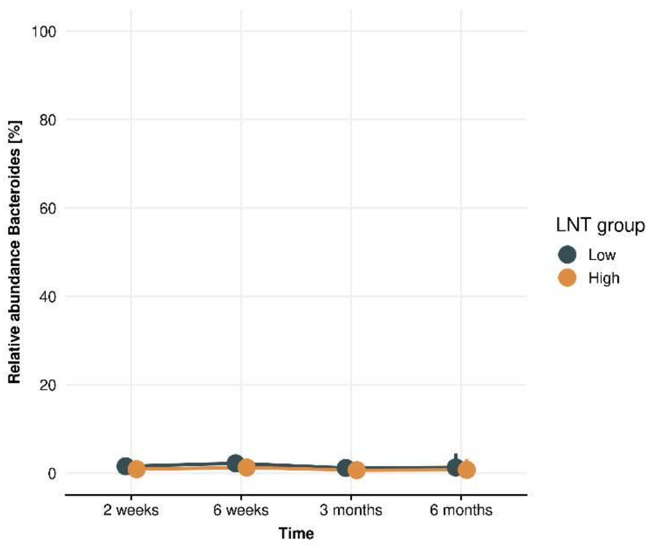

c)

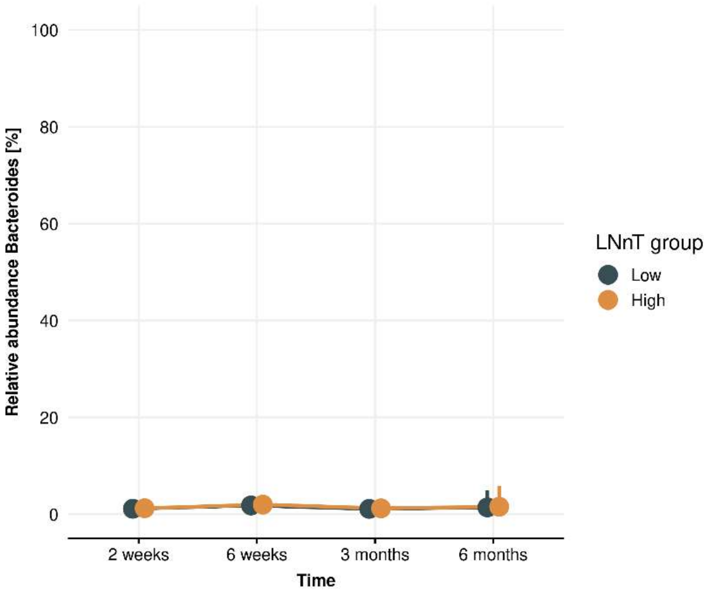
100
80
60
40
20
0
Relative abundance Bacteroides [%]
2 weeks
6 weeks
3 months
6 months
Time
LNnT group
Low
High

d)

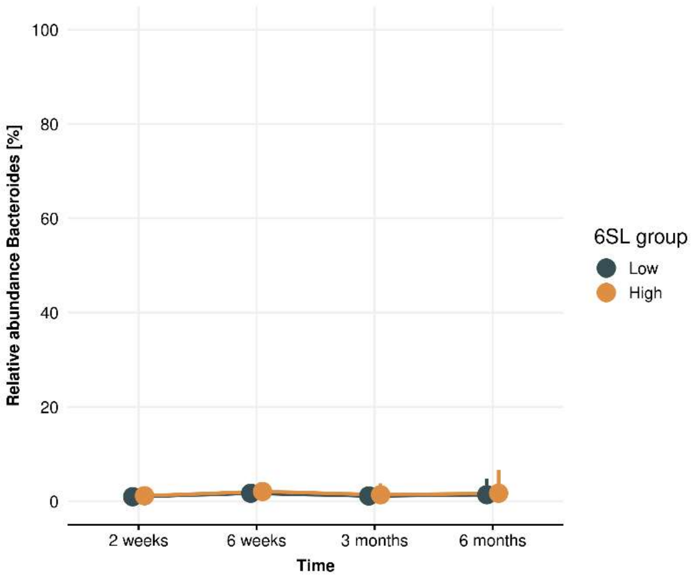
100
80
60
40
20
0
Relative abundance Bacteroides [%]
2 weeks
6 weeks
3 months
6 months
Time
6SL group
Low
High

Suppl Figure Legends

Supplementary Figure 1. Genus (a) and species-level (b) infant stool microbiome composition stratified by feeding module (breastfeeding duration <3, 3-6, >6 months) at each time point (infant age 2 weeks, 6 weeks, 3 months, 6 months). Values are means across all infants in a feeding module group. Only the 15 most abundant taxa are shown.

Supplementary Figure 2. Lactobacillus and Lacticaseibacillus abundance in infant stool microbiota stratified by feeding module (breastfeeding duration) at each time point (infant age). Values are means across all infants in a feeding module group.

Supplementary Figure 3. Effects of infant age and higher Parity (2 or more) on phylum-level microbiome composition across all time windows (infant age). Only significant effects after FDR adjustment are shown.

Supplementary Figure 4. *Bifidobacterium* levels across time (infant age 2 weeks, 6 weeks, 3 months, 6 months) in high and low a) LNT, b) LNnT, c) 3'SL and d) 6'SL groups. Plots show gLMM estimates (95% confidence intervals) of *Bifidobacterium* relative abundance in infants with either low or high HMO levels (where 'low' or 'high' is defined as lower or higher than median levels at each time point). LNT, LNnT, 3'SL and 6'SL were not associated with *Bifidobacterium* relative abundance.

Supplementary Figure 5. *Bacteroides* levels across time (infant age 2 weeks, 6 weeks, 3 months, 6 months) in high and low a) LNFP1 b) LNT, c) LNnT, d) 6'SL groups. Plots show gLMM estimates (95% confidence intervals) of *Bacteroides* relative abundance in infants with either low or high HMO levels (where 'low' or 'high' is defined as lower or higher than median levels at each time point). The models for 2'FL and 3'SL levels did not converge. None of the HMOs were associated with *Bacteroides* relative abundance.
