# Supplementary material for: Early development of infant gut microbiota in relation to breastfeeding and human milk oligosaccharides
Source: Front Nutr. 2023 Mar 9;10:1003032. doi: 10.3389/fnut.2023.1003032 (PMC10034312; doi:10.3389/fnut.2023.1003032)
Supplement: Supplementary file 2 [file Presentation_1.pdf]

Supplementary Methods

DNA extraction from stool samples

Fecal samples were first thawed at 4 °C. Next, in a 2.0 mL screw-cap tube containing 0.5 g of 0.1 mm sterilized zirconia beads, approximately 250 mg of faeces and 700 µL S.T.A.R. buffer (Roche, Indianapolis, IN, USA) were added. The FastPrep instrument (MP Biomedicals, Santa Ana, CA, USA) was used for lysis at 5.5 m/s for 3 times 1 min at room temperature. Samples were incubated, shaking at 100 rpm and 95 °C for 15 min. The samples were then centrifuged at 16000 g for 5 min at 4 °C. The collected supernatant was kept on ice, while another lysis round as described above, except that only 350 µl S.T.A.R. buffer was added, was done with the remaining stool pellet. The supernatant kept on ice was then pooled with the supernatant from the second lysis round. Purification of DNA was performed on the automated Maxwell instrument (Promega, Madison, WI, USA) by applying the Maxwell 16 Tissue LEV Total RNA Purification Kit (Promega) according to the manufacturer's instructions. To the first well of the Maxwell cartridge 250 µL of the supernatant was added and finally DNA was eluted with 50 µL of RNAse/DNAse free water.

PCR amplification of 16S rRNA gene in stool DNA, library preparation and sequencing

Using a 2-step PCR, barcoded amplicons from the V3–V4 region of 16S rRNA genes were generated. For initial amplification of the V3–V4 part of the 16S rRNA universal primers with the following sequences were used: forward primer, '5-TCGTCGGCAGCGTCAGATGTGTATAAGAGACAGCCTACGGGAGGCAGCAG'; reverse primer, '5-GTCTCGTGGGCTCGGAGATGTGTATAAGAGACAGTACNVGGGTATCTAAKCC', appended with Illumina adaptor sequences. The PCR amplification mixture contained: 1 µL

faecal sample DNA, 1 µL barcoded forward primer (10 µM), 14 µL master mix (1 µL KOD Hot Start DNA Polymerase (1 U/µL; Novagen, Madison, WI, USA), 5 µL KOD-buffer (10×), 3 µL MgSO4 (25 mM), 5 µL dNTP mix (2 mM each)), 1 µL (10 µM) of reverse primer and 33 µL sterile water (total volume 50 µL). PCR conditions were: 95 °C for 2 min followed by 30 cycles of 95 °C for 20 sec, 55 °C for 10 sec, and 70 °C for 15 sec. 500 bp PCR amplicons were then purified using the MSB Spin PCRapace kit (Invitek, Berlin, Germany). PCR products were purified, and quantified followed by multiplexing, clustering and sequencing on an Illumina MiSeq with the paired-end (2x) 300 bp protocol and indexing. The sequencing run was analyzed using Illumina CASAVA pipeline (v1.8.3) with de-multiplexing based on sample-specific barcodes. The sequence reads of too low quality (only "passing filter" reads were selected) were removed.

Bioinformatics pipeline

Raw sequencing data was quality controlled using FastQC v0.11.9 (Andrews et al., 2010) using vsearch v2.18.0 (Rognes et al., 2016). After trimming the primers, the forward and the reverse reads were merged using a permissive threshold (i.e. allowing up to 30 differences in the overlapping region) discarding merged sequences shorter than 250 bp or longer than 500 bp. Remaining sequences were filtered allowing at most 1 expected error per sequence and no ambiguous bases. Amplicon Sequence Variants (ASVs) were inferred from dereplicated filtered sequences using the UNOISE3 algorithm (Edgar, 2016b). Chimeric ASVs were removed using the '--uchime3_denovo' vsearch command (Edgar, 2016a). Taxonomy was assigned to the ASVs using the DADA2 R package v1.18.0 (Callahan et al., 2016) and the SILVA 16S rRNA database v138.1. Species-level taxonomy was assigned using exact matches and allowing for at most 3 multiple species assignments. ASVs were aligned using MAFFT v7.487 (Katoh & Standley,

2013) to obtain a multiple sequence alignment from which a phylogenetic tree was constructed using FastTreee v2.1.11 double precision using a generalized time-reversible model of nucleotide evolution (Price et al., 2010). The tree was midpoint-rooted using phytools v0.7.70 (Revell, 2012). Merged sequences were mapped against the non-chimeric ASVs to construct a per-sample ASV table. The ASV table, taxonomy, phylogenetic tree and ASV sequences were integrated using the phyloseq v1.34.0 R package (McMurdie & Holmes, 2013). The bioinformatic pipeline was implemented using snakemake v6.8.0 (Mölder et al., 2021). The raw sequencing data has been deposited to the European Nucleotide Archive (study accession PRJEB50418).

Data Analysis

A multilevel Principal Component Analysis (PCA) was performed to visualize within-infant variance on the rarefied center log-ratio (CLR)-transformed counts using the 'infant' term for the multilevel decomposition for repeated measurements. A pseudocount of 1 was added to all sample counts prior to the CLR-transformation and PCA using the mixOmics v6.17.26 R package (Rohart et al., 2017).

An additional offset term (log(Library size)) was added to all gLMMs models having taxa as outcomes in order to account for differences in library size between samples. In gLMMs used to examine the effect of individual HMOs on Bifidobacterium abundance the respective HMO was added as an additional fixed effect. Separate models were fitted with secretory status as an additional fixed effect. A gaussian distribution was used to model alpha-diversity measures and HMO concentrations while unrarefied taxa counts (at different taxonomic levels: species, genus, family, and phylum) were modeled using a negative binomial (nbinom1) distribution with a zero-inflated component.
